# A Virtual Supermarket Program for the Screening of Mild Cognitive Impairment in Older Adults: Diagnostic Accuracy Study

**DOI:** 10.2196/30919

**Published:** 2021-12-03

**Authors:** Mingli Yan, Huiru Yin, Qiuyan Meng, Shuo Wang, Yiwen Ding, Guichen Li, Chunyan Wang, Li Chen

**Affiliations:** 1 School of Nursing Jilin University Changchun China; 2 Senior Officials Inpatient Ward First Hospital of Jilin University Changchun China

**Keywords:** virtual reality, mild cognitive impairment, dementia, ambient intelligence, digital health, elderly population, aging

## Abstract

**Background:**

Mild cognitive impairment (MCI) is often a precursor of dementia, and patients with MCI develop dementia at a higher rate than healthy older adults. Early detection of cognitive decline at the MCI stage supports better planning of care and interventions. At present, the use of virtual reality (VR) in screening for MCI in older adults is promising, but there is little evidence regarding the use of virtual supermarkets to screen for MCI.

**Objective:**

The objectives of this study are to validate a VR game–based test, namely, the Virtual Supermarket Program (VSP), for differentiating patients with MCI and healthy controls and to identify cutoff scores for different age levels.

**Methods:**

Subjects were recruited from several nursing homes and communities in Changchun, China. They were divided into a healthy control group (n=64) and an MCI group (n=62). All subjects were administered the VSP and a series of neuropsychological examinations. The study determined the optimal cutoff, discriminating validity, concurrent validity, and retest reliability of the VSP. We used the area under the receiver operating characteristic curve (AUC) to evaluate the discriminating validity and obtain the optimal cutoff values. Pearson correlation analysis and the intraclass correlation coefficient were used to evaluate the concurrent validity and retest reliability, respectively.

**Results:**

A cutoff score of 46.4 was optimal for the entire sample, yielding a sensitivity of 85.9% and specificity of 79.0% for differentiating individuals with MCI and healthy controls, and the AUC was 0.870 (95% CI 0.799-0.924). The median index of VSP score was 51.1 (range 42.6-60.0). There was a moderate positive correlation between the VSP total score and Mini-Mental State Examination score (*r*=0.429, *P*<.001). There was a strong positive correlation between VSP total score and Montreal Cognitive Assessment score (*r*=0.645, *P*<.001). The retest reliability of the VSP was feasible (*r*=0.588, *P*=.048).

**Conclusions:**

The VSP is interesting and feasible for subjects. It shows high sensitivity and specificity for the identification of MCI in older adults, which makes it a promising screening method. The VSP may be generalized to older adults in other countries, although some cultural adaptation may be necessary.

**Trial Registration:**

Chinese Clinical Trial Registry ChiCTR2000040074; https://www.chictr.org.cn/showprojen.aspx?proj=64639

## Introduction

Dementia is becoming more prevalent worldwide. According to the “World Alzheimer Report 2018” [1], 50 million people worldwide had dementia in 2018. As the most populous country, China has experienced the greatest burden of dementia. The total number of people with dementia in China will reach 23.3 million by 2030 [2], and in 2020, the burden of dementia-related disability and care in China reached US $250 billion, accounting for nearly one-fifth of the global dementia-related costs [3]. However, there is no effective treatment for dementia; therefore, increasing attention has been given to the precursors of dementia. Mild cognitive impairment (MCI) is often a precursor of dementia, but it can also be due to other pathologies (such as Parkinson disease and tumors) [4,5]. Although not all forms of MCI lead to dementia, the mean annual conversion rate of MCI to dementia is approximately 10%, which is far higher than the annual incidence (1%-2%) in healthy older adults [6,7]. Relevant studies have shown that interventions for MCI can effectively delay or even reverse cognitive decline [8]. Therefore, early detection of cognitive decline at the MCI stage is vital for better planning of care and interventions. 

The commonly used cognitive screening methods are paper-and-pencil neuropsychological testing [9] and computerized cognitive testing [10,11]. Compared to neuropsychological testing, the advantages of computerized cognitive testing include standardized administration procedures and presentation of stimuli and accurate measurement of response time [12,13]. Virtual reality (VR) is widely used in research with computerized cognitive testing for MCI [14,15]. VR is an ecologically valid and informative evaluation instrument and provides an opportunity to improve cognitive screening [16-18]. Studies have shown that VR is recommended for the screening of older adults with MCI or dementia [15,19]. Among older adults with cognitive impairment screened through VR, virtual supermarket has been accepted [20] and could be used to detect cognitive impairment in older adults [21,22].

Virtual supermarkets are a novel method for screening for MCI and assessing cognition through shopping-related activities. Related research using virtual supermarkets is effective in screening for MCI [20,22,23]. Atkins et al [24] developed the Virtual Reality Functional Capacity Assessment Tool, which assesses functional abilities related to shopping, and found a significant relationship between task performance and cognitive performance. Werner et al [22] developed the Virtual Action Planning-Supermarket, which allows subjects to navigate the store, purchase 7 items, and then pay for them, and they distinguished healthy older adults and MCI patients. Significant differences were found between healthy older adults and patients with MCI in the Virtual Action Planning-Supermarket measures. However, they assessed few cognitive domains and screened for cognitive impairment by assessing only executive function. Zygouris et al [20] developed a screening tool called the Virtual Supermarket Test to detect MCI and assessed 4 cognitive abilities in older adults through four shopping-related tasks. The program can operate on Android-based tablet devices or computers. The discriminative validity of the program was good in older adults over 70 years of age. However, Zygouris et al [20] developed virtual supermarket as a cognitive training application that does not specifically screen for MCI, and it screens few cognitive domains. Nevertheless, the literature supports virtual supermarket in screening for MCI [23]. However, the virtual supermarkets developed in other countries may not be suitable screens for older Chinese adults, and there is no virtual supermarket software to screen for MCI in China.

The Virtual Supermarket Program (VSP) developed in this study screened subjects with MCI by assessing their ability to shop in conditions relevant to daily life. Subjects could actively explore the VSP environment while performing the task. Compared with other versions of the virtual supermarket, VSP provides a more comprehensive screening, involves a rich environment with VR technology, and is more in line with the living and cultural habits of older adults in China. Therefore, we aimed to verify the feasibility of VSP for screening for MCI in older adults in China.

## Methods

### Study Design

We evaluated the feasibility of VSP for screening patients with MCI (diagnosed in accordance with Petersen criteria) [25] over 60 years of age in China. The study followed the STARD checklist for the reporting of studies of diagnostic test accuracy [26]. This study was conducted between December 2020 and February 2021. The study protocol was approved by the School of Nursing (approval 2020082805), Jilin University, and it has been registered in the Chinese Clinical Trial Registry (registration ChiCTR2000040074). All subjects were informed about the purpose of the study before providing their verbal consent. The VSP used in this study was a new tool developed by the authors' team at the School of Nursing, Jilin University, China, and a company was entrusted to provide technical support.

### Population

Subjects were from several nursing homes and communities in Changchun, China. The inclusion criteria of the subjects with MCI were as follows: (1) MCI diagnosis in accordance with the Petersen criteria [25]; (2) age≥60 years; (3) a Montreal Cognitive Assessment (MoCA) scale [27] score of <26; (4) a Mini-Mental State Examination (MMSE) [28] score of ≥24; (5) no impairment in functional daily living activities (Activities of Daily Living [ADL] scale [29] score of ≤26); (6) absence of psychiatric illnesses, with particular reference to depressive symptoms (15-item Geriatric Depression Scale [GDS-15] [30] score of ≤8); and (7) absence of severe auditory/visual loss that can prevent the use of technological devices and from executing the VSP. The inclusion criteria of subjects in the healthy control group were inclusion criteria (2) and (4)-(7) from the aforementioned list and an MoCA score of ≥26.

### Sample Size

We calculated the sample size using equation 1 [31] 
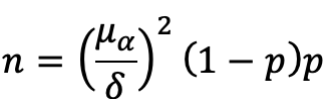
, and we set the confidence level (1-*α*) at 95% and the allowable error (δ) of sensitivity and specificity at 10%. We estimated the sample size required for sensitivity (the sample size for the MCI group) and the sample size required for specificity (the sample size for the healthy control group).

The sensitivity and specificity of the tools developed by Zygouris et al [21] were 82.35% and 95.24%, respectively, which were used to calculate the sample size for the MCI group and healthy control group in this study. We predicted a dropout rate of 10%. The sample size of the MCI group was 62, and that of the healthy control group was 20 in this study. Therefore, we needed to enroll a minimum of 82 subjects.

### Study Design

This study recruited subjects over the age of 60 years in nursing homes and communities. The subjects who volunteered for the study were first administered a neuropsychological test to screen for MCI in accordance with the Peterson criteria [25]. The test took place in a quiet and bright room. First, baseline characteristics (age, education level, height and weight, and previous computer use before VSP) and past medical history were collected. Older adults with depression were excluded using the GDS-15. Then, the Clinical Dementia Rating (CDR) and MMSE were used to exclude patients with dementia. Thereafter, MoCA and functional status (ADL scale) measures were collected. Last, VSP tests were performed for all subjects. Prior to each task in the VSP, the subjects were repeatedly instructed on the task, and operation instructions were available throughout the test through the user interface. The VSP was managed by independent researchers, who were blinded to the subjects' cognitive status. All subjects used the VSP application under the same circumstances.

**Figure 1:**
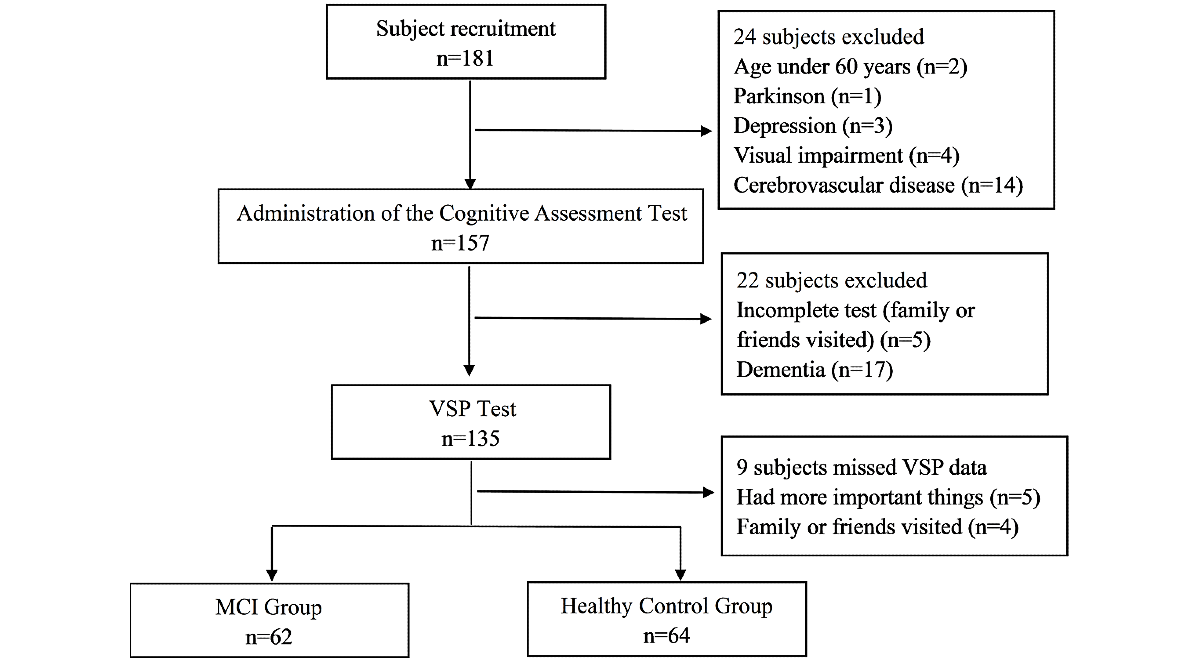
Flowchart of subject recruitment and testing. MCI: mild cognitive impairment; VSP: Virtual Supermarket Program.

All data were obtained directly from the subjects and included neuropsychological test scores, VSP scores, and general information. The assessors were blinded to the cognitive status or grouping of the subjects. Figure 1 shows a flowchart of subject recruitment and testing.

### Neuropsychological Assessments

Subjects were administered a battery of neuropsychological tests, including the CDR, MMSE, GDS-15, ADL, and MoCA. The reference standard results were available to the testers. The neuropsychological tests used in this study were as follows.

#### CDR

The CDR assesses 6 abilities on a 5-point scale [32]: no dementia (CDR=0), questionable dementia (CDR=0.5), mild dementia (CDR=1.0), moderate dementia (CDR=2.0), and severe dementia (CDR=3.0). Finally, the results of the 6 ability ratings are combined into one rating on the basis of the scoring standard, and there is no dementia when the CDR was zero [33].

#### MMSE

The MMSE has 30 questions, and the highest potential score is 30. The optimal cutoff values differ for subjects with different educational levels. Subjects who are illiterate and scored ≤17 points, those with a primary school education who scored ≤20 points, and those with a high school education or above who scored ≤24 points are classified as having dementia [34].

#### GDS-15

The GDS-15 comprises 15 questions to which subjects are asked to respond with “yes” or “no.” The total score can range from 0 to 15, and a total score of >8 is associated with depressive symptoms [30].

#### MoCA Scale

The MoCA scale comprises 32 questions, and the highest potential score is 30. If the number of years of education is ≤12 years, 1 point is added to the actual total score, and a final total score of >26 is considered normal [27].

#### ADL Scale

The ADL scale comprises 14 items that are scored at 4 levels. The total score can range from 14 to 56, and a total ADL score of >26 points indicate an impaired ability to perform activities of daily living [29].

### Virtual Supermarket Program

We developed the VSP to be in line with Chinese cultural habits and with reference to the virtual supermarket of Zygouris et al [20,21]. We mimicked the MoCA and designed the VSP with VR technology. For instance, for the assessment of temporal orientation, the MoCA scale asks the subjects to identify the current date. In the VSP, we designed the link to input the payment password, and the password was the current date. All the tasks in the VSP were designed in this manner. Shopping-related tasks were used to assess learning and memory, executive functions, language, time orientation, and complex attention of older adults and to explore the feasibility of the VSP for screening MCI in older Chinese adults. There were 9 tasks in the VSP, and it was run on a computer (CPU, i5 or higher; memory, ≥16 GB; independent video memory, ≥2 GB; storage capacity, ≥200 GB) for which subjects did not need VR glasses and a handle to operate. The VSP was operated with a computer mouse and a keyboard (Multimedia Appendix 1). The following tasks were included in the VSP: task 1: memorize shopping list; task 2: look at the map to get directions to the supermarket and make purchases on the list; task 3: recall the sales announced by voice in the supermarket; task 4: label the item that have lost their labels; task 5: determine whether the correct items were purchased; task 6: calculate the amount for the purchased items; task 7: enter the correct payment code (current date); task 8: compare lucky draw numbers at the service center; and task 9: choose the correct bus route home.

The VSP assessed five cognitive domains [35]: learning and memory (tasks 1 and 3), executive functions (tasks 2, 6, and 9), language (tasks 4 and 5), time orientation (task 7), and complex attention (task 8). The VSP score ranges from 0 to 60 (Multimedia Appendix 2). The technician devised a fixed algorithm to calculate the score for each task and the total VSP score on the basis of the accuracy with which the subject completed the task. There was no time limit for subjects to perform each VR task.

### Statistical Analysis

Statistical analyses were performed using SPSS Statistics (version 23.0, IBM Corp) and MedCalc (version 19.2, MedCalc Software Ltd). The normality of data distributions was assessed using the Shapiro–Wilk test. Mean (SD) values were used to describe continuous variables that were normally distributed, and median (IQR) values were used to describe the continuous variables that were not normally distributed. Descriptive statistics included n (%) values for categorical variables. Independent samples *t* tests were used to compare continuous variables that were normally distributed between the two groups, nonparametric Mann-Whitney *U* rank sum tests were used to compare continuous variables that were not normally distributed between the two groups, and chi-square tests were used for the categorical variables. The feasibility of VSP screening for MCI was verified by measures of optimal cutoff, discriminating validity, concurrent validity and test-retest reliability for the VSP. Discriminating validity was examined through receiver operating characteristic (ROC) analysis of VSP total scores. The area under the receiver operating characteristic curve (AUC) can range between 0 and 1. The closer the AUC to 1, the better the diagnosis [36]. Concurrent validity was examined through Pearson correlation analyses of VSP total scores against MMSE scores [34] and MoCA scores [27]. Test-retest reliability was assessed with an intraclass correlation coefficient (ICC). A 2-sided *P* value of <.05 indicated statistical significance.

## Results

### Demographics and Characteristics by Cognitive Status

The demographics and cognitive characteristics of the subjects are presented in Table 1. In the actual process of recruitment, the healthy control group collected more than the minimum sample size we previously calculated. Considering that a large number of samples can improve the test efficiency, we expanded the sample size. Initially, 181 participants were recruited (Figure 1). No adverse events occurred during the study. Finally, the study included 126 older adults (45 male and 91 female). The mean age was 77.12 years (range 61-94 years), and years of education ranged from 0 to 16 years. In total, 62 older adults were enrolled in the MCI group and 64 were enrolled in the healthy control group. There was a significant difference in age between the 2 groups (*P*=.02). No between-group differences in sex distribution (*P*=.06), years of education, or computer experience (having used a computer at least once) were found (*P*=.08). As expected, the 2 groups had significantly different MMSE scores and MoCA scores (*P*<.001).

**Table 1:** Demographics and cognitive characteristics of subjects (N=126).

Characteristics		Healthy controls (n=64)	Patients with mild cognitive impairment (n=62)	*T*, *z*, or chi-square test	*P* value
Age (years), mean (SD)		73.5 (16.0)	82.00 (15.50)	–2.486^a^	.01
**Sex, n (%)**				3.613^b^	.06
	Female	13.0 (20.3)	22.0 (35.5)		
	Male	51.0 (79.7)	40.0 (64.5)		
**Years of education, n (%)**				6.875^b^	.08
	Primary school	10.0 (15.6)	4.0 (6.5)		
	Middle school	13.0 (20.3)	19.0 (30.6)		
	High school	13.0 (20.3)	20.0 (32.3)		
	University	28.0 (43.8)	19.0 (30.6)		
**Computer experience ( having used a computer at least once), n (%)**				2.873^b^	.09
	Yes	29.0 (45.3)	19.0 (30.6)		
	No	35.0 (54.7)	43.0 (64.9)		
**Cognitive assessment score, mean (SD)**					
	Mini-Mental State Examination score (maximum score=30)	28.5 (1.3)	27.0 (1.6)	5.668^c^	<.001
	Montreal Cognitive Assessment score (maximum score=30)	26.6 (1.0)	20.8 (2.7)	16.106^c^	<.001

^a^Mann-Whitney *U* test.

^b^Chi-square test.

^c^Independent samples *t* tests.

### Performance in the VSP Between Patients With MCI and Healthy Controls

The performance scores for the individual tasks on the VSP are summarized in Table 2. The mean scores in each VSP task in the healthy control group were higher than those in the MCI group. With the exception of the difference for task 6, these differences were significant. The total VSP score in the MCI group was significantly lower than that in the healthy control group (*P*<.001).

**Table 2:** Performance scores in the Virtual Supermarket Program.

Task score	Healthy controls (n=64), mean (SD)	Patients with mild cognitive impairment (n=62), mean (SD)	*t* test (independent samples)	*P* value
Task 1	9.9 (0.5)	9.6 (1.0)	1.999	.048
Task 2	4.0 (2.0)	2.3 (2.5)	4.265	<.001
Task 3	6.0 (3.5)	4.1 (3.8)	2.940	.004
Task 4	6.0 (0.5)	5.6 (1.2)	2.087	.04
Task 5	5.6 (0.8)	4.5 (2.0)	4.379	<.001
Task 6	5.8 (0.9)	5.8 (1.1)	0.213	.83
Task 7	5.5 (1.6)	4.7 (2.5)	2.328	.02
Task 8	4.0 (1.4)	2.0 (1.6)	7.407	<.001
Task 9	4.8 (1.1)	2.8 (2.5)	5.708	<.001
Total	51.4 (4.8)	41.3 (7.7)	8.894	<.001

### Potential of the VSP to Distinguish Between Patients With MCI and Healthy Controls

**Figure 2:**
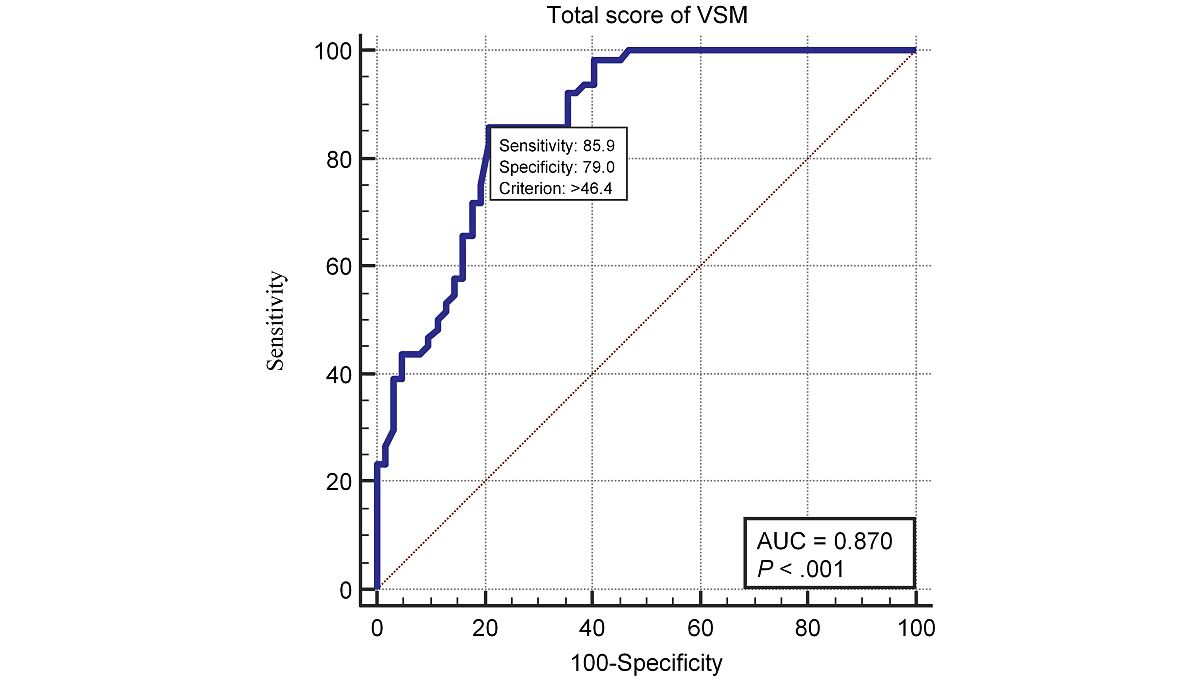
Receiver operating characteristic curve analysis for the total score in the Virtual Supermarket Program.

An ROC curve analysis was performed with the total VSP scores that were used to distinguish between patients with MCI and healthy controls (Figure 2). The AUC was found to be 0.870 (95% CI 0.799-0.924, *P*<.001). An optimal statistical cutoff was achieved at a cutoff score of 46.4 points (85.9% sensitivity, 79.0% specificity).

**Figure 3:**
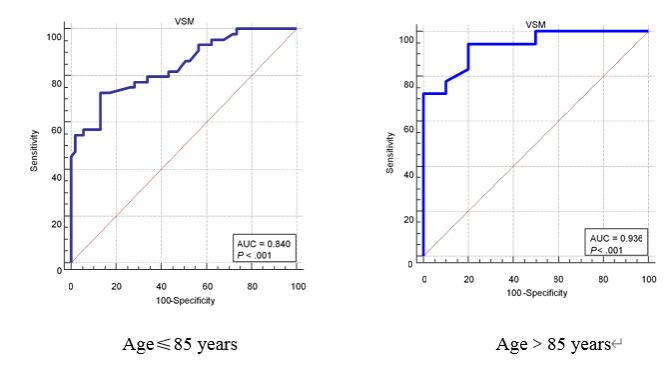
Receiver operating characteristic curve analysis for the total score in the Virtual Supermarket Program among different age groups.

Among subjects aged under 85 years, the optimal cutoff was 46.5 points, and the sensitivity and specificity were 72.73% and 86.79%, respectively (AUC=0.840, *P*<.001). Among the subjects aged over 85 years, the optimal cutoff was 47.3 points, yielding a sensitivity of 94.4% and specificity of 80.0% (AUC=0.936, *P*<.001). The ROC curve stratified by age is shown in Figure 3, and the discriminative validity is shown in Table 3.

**Table 3:** The total score in the Virtual Supermarket Program and the discriminant results of age stratification.

		Optimal cutoff	Sensitivity, %	Specificity, %	Area under the receiver operation characteristic curve	95% CI	*P* value	Youden index J
Entire sample		46.4	85.90	79.00	0.870	0.799-0.924	<.001	0.650
**Age (years)**								
	≤85	46.5	72.73	86.79	0.840	0.752-0.907	<.001	0.600
	>85	47.3	94.44	80.00	0.936	0.775-0.993	<.001	0.744

### Correlations Between the VSP and Routine Cognitive Screening Assessments

There was a moderate positive correlation between the total VSP scores and MMSE scores (*r*=0.429, *P*<.001). There was a strong positive correlation between the total VSP scores and MoCA scores (*r*=0.645, *P*<.001). In addition, we found a negative correlation of the total VSP scores with age (*r*=–0.308, *P*<.001). Table 4 shows the correlation matrix between the different variables.

**Table 4:** Correlation matrix demonstrating the degree of associations among the Mini-Mental State Examination score, Montreal Cognitive Assessment score, age, education, and total performance scores.

Variable		Age	Education	Mini-Mental State Examination Score	Montreal Cognitive Assessment score	Score in the Virtual Supermarket Program
**Age**						
	*r*	—^a^	–0.119	–0.342	–0.247	–0.308
	*P* value	—	0.186	<.001	.005	<.001
**Education**						
	*r*	–0.119	—	0.269	0.110	0.098
	*P* value	.19	—	.002	.22	.27
**Mini-Mental State Examination score**						
	*r*	–0.342	0.269	—	0.592	0.429
	*P* value	<.001	.002	—	<.001	<.001
**Montreal Cognitive Assessment score**						
	*r*	–0.247	0.110	0.592	—	0.645
	*P* value	.005	0.22	<.001	—	<.001
**Score in the Virtual Supermarket Program**						
	*r*	–0.308	0.098	0.429	0.645	—
	*P* value	<.001	0.27	<.001	<.001	—

^a^—: not determined.

### Retest Reliability of the VSP

A month after the first test, 9 healthy controls were randomly selected to receive the VSP again, and the results showed that the ICC between total VSP scores was feasible (*r*=0.588, *P*=.048).

## Discussion

### Principal Findings

To our knowledge, this is the first study to use a locally developed virtual supermarket task to screen for MCI among older adults in China. The VSP we designed in this study was a computerized application running on a computer to screen for MCI. Through verification of its screening effectiveness, the VSP had the highest sensitivity and specificity when the optimal cutoff was 46.4. The VSP can effectively distinguish older adults with MCI from healthy controls, and it was more suitable for older adults aged 85 years and above. Performance in the VSP had a strong positive correlation with MoCA scores but a moderate positive correlation with MMSE scores. The retest reliability of the VSP scores was feasible.

### Comparing Performance Between Patients With MCI and Healthy Controls

We found that the overall scores and scores for each task were significantly higher in the healthy control group, but task 6 did not show a significant difference between the 2 groups. In task 6, the subjects were asked to calculate the total cost of the items they had purchased. Given the situation of older adults, the settings related to the task list in the system were set to be relatively simple, which is probably why task 6 did not show a significant difference between the MCI subjects and healthy controls. This is a solvable problem. In the future, we will adjust the difficulty of task 6 by increasing the number of items purchased or adjusting the price of items.

Compared to the healthy controls, the subjects in the MCI group performed worse on tasks (tasks 1, 2, 3, 5, and 9) that involved learning and memory and executive function. The MCI subjects were more likely to forget or buy the wrong item on their shopping list, and they could not perform the shopping tasks very well. This is evidence that these tasks were better at distinguishing between the 2 groups. Task 5 and task 8 represent the ingenuity in our task design. Task 5 involved an assessment of learning and memory, executive function and language that comprehensively evaluated the subjects’ cognition. Task 8 evaluated the subjects’ complex attention. The subjects needed to use their attention, hearing, and finger reflexes at the same time to complete the task. This task could distinguish the MCI subjects and healthy controls well. Above all, these tasks highlight the effectiveness of the VSP in distinguishing between the MCI subjects and healthy controls.

### Discriminating the Validity of the VSP Total Score

In this study, the VSP score could effectively distinguish between MCI subjects and healthy controls. The sensitivity and specificity were 85.90% and 79.00%, respectively. The AUC based on the VSP scores was 0.870 in this study. Compared with other studies, this level of accuracy is relatively high. Boz et al [23] used the Virtual Supermarket, which was developed in Greece [21], to screen for MCI among older Turkish adults. They reported that the sensitivity and specificity were 74.00% and 85.00%, respectively; they did not report the AUC. The sensitivity and specificity of the virtual supermarket task were better in our study than in that by Boz et al [23]. This may have resulted from using Euros on the payment screen, which adds additional complexity and cognitive burden [23]. The system of Cognitive Assessment by Virtual Reality [35] is adapted from the RE@CH module, and Chua et al [37] reported an AUC of 0.821, a sensitivity of 78.2%, and a specificity of 75.7% in the Singaporean population. Compared to the task used by Chua et al [37], the VSP is more in line with the culture and living habits of older Chinese adults, and the sample size in our study was larger.

Age is an important factor in cognition. Among subjects younger than 85 years, the AUC, sensitivity, and specificity were 0.840, 72.73%, and 86.79%, respectively. Among subjects over 85 years of age, the AUC, sensitivity, and specificity were 0.903, 94.44%, and 80.00%, respectively. As people age, their cognitive performance declines. Compared with younger subjects, older subjects committed more task errors and had a harder time concentrating [38]. This may have led to a better discriminative validity of the VSP among individuals over 85 years of age. This finding indicates that our VSP has a wider range of uses and is suitable for older adults of different ages.

### Associations Among VSP Scores, Age, Education, and Neuropsychological Testing

VSP scores were negatively correlated with age, which is consistent with the work of Chua et al [37,39]. Considered a task involving multiple demands, the VSP was formed by a series of activities consisting of goals during its execution, and it requires activation and cooperation of multiple cognitive domains [40]. The older the subjects, worse the VSP performance and scores. There was no correlation between VSP scores and education level. The literature is not conclusive regarding the impact of education level on cognition [41,42]. The VSP that was developed and designed in this study was suitable for older adults with different educational levels and was intended to have wide applicability. The tasks closely reflect to the habits of older adults, and successful performance does not necessarily depend on education. Kang et al [43] did not report an effect of education levels among older adults with a multiple-order VR task for neuropsychological assessment.

The total VSP performance scores showed a moderate positive correlation with MMSE scores (*r*=0.429, *P*<.001) and a strong positive correlation with MoCA scores (*r*=0.645, *P*<.001). The correlation between VSP scores and MoCA scores was significantly higher than that between VSP scores and MMSE scores, which was consistent with our expectation. The VSP used in this study was specifically developed for detecting MCI, and the design of VSP mimicked the MoCA scale. Research has shown that the MoCA scale has more advantages than the MMSE scale in detecting visual executive dysfunction [44]. Therefore, the correlation between VSP and MoCA scores may be stronger than that between VSP and MMSE scores. In addition, although the MMSE has a modest specificity for screening MCI, its sensitivity for screening MCI is low, and the capacity to detect MCI converters is poor [28]. This outcome is consistent with that of Oliveira et al, who also showed a weak correlation between MMSE scores and performance in VR research [39]. Chan et al [45] and Chua et al [37] also conducted correlation analyses with MoCA scores when verifying the screening application for MCI, which they had developed, and showed strong correlations between the measures.

### Clinical Implications

Our findings provide preliminary evidence that the VSP can be used for MCI screening in Chinese communities and nursing homes, providing a more convenient, concise, and effective tool for MCI screening in China. We introduced several short activities based on VR to assess different cognitive domains, rather than using a single game, which allowed us to assess impairments in the major cognitive domains. The subjects were screened using shopping tasks based on the abovementioned cognitive domains. After completing the task, the obtained scores were compared with the cutoff value of 46.4, which could preliminarily screen whether the subjects had MCI. This screening program could provide rapid screening in Chinese communities and nursing homes. It expands this new method of screening for MCI and provides new ideas and methods for the development of MCI screening software in China. In addition, compared to traditional neuropsychological tests, the VSP has an automatic scoring function and allows data to be extracted and interpreted more easily. The VSP has other advantages, including administration cost savings and better ecological relevance, and is suitable for unsupervised use in a home or clinical setting. We observed that owing to the visual content and interaction style through VR, most subjects found the task more interesting than traditional screening tools and were more willing to take the test. Yun et al [46] also reported elderly people's interest in VR. Implementation studies are needed in the future to evaluate its rollout in clinical practice.

### Study Limitations and Future Research

There are several limitations in the study, which limit the generalizability of the results. First, subjects in the MCI group were older than those in the healthy control group, which may have affected their performance. Age is an important factor in cognition. To further evaluate the use of VSP in cognitive assessment, demographic data matching and expanding the sample size can be incorporated in future studies.

Second, even if some of the subjects had previously used a computer, many of the subjects had limited familiarity with new technologies, particularly computer equipment and operations. Oliveira et al [39] showed that experience with the computer was not a relevant predictor for any of the models investigated. We also need to further verify the impact of new technology on the ability of older adults to engage in the task.

Third, the correlations between VSP subtests assessing specific cognitive domains and existing neuropsychological tests that assess the same domains were not verified. In future research, we need to verify the correlation between the two to verify the feasibility of VSP subtests for assessing specific cognitive domains.

Owing to technical limitations, this study did not verify the feasibility of the time to complete the VSP in screening for MCI. In the future, it will be necessary to improve the assessment technology of VSP stay time in each area to verify the feasibility of the total time of VSP to detect older adults with MCI. In addition, we need to further verify the retest reliability of the VSP in future research. We tested the VSP again a month after the first test, but the community and nursing home were closed owing to the COVID-19 pandemic, and we could not enter and leave freely. Finally, only nine subjects were retested on the VSP. In the future, it will be necessary to further expand the sample size to verify the retest reliability of the VSP.

### Conclusions

The VSP we designed in this study is a computerized application running on a computer to identify MCI. This study demonstrated the feasibility of the VSP in distinguishing between MCI and healthy adults among older Chinese adults. The results of this feasibility study are invaluable. Future studies should focus on testing and validating longitudinal data for the ability to track the progression of cognitive decline.

## Appendix

Multimedia Appendix 1 Screenshots of the VSP.

Multimedia Appendix 2 VSP score of each task and total score.

## Abbreviations

ADL: Activities of Daily Living
CDR: Clinical Dementia Rating
GDS-15: 15-item Geriatric Depression Scale
ICC: intraclass correlation coefficient
MCI: mild cognitive impairment
MMSE: Mini-Mental State Examination
MoCA: Montreal Cognitive Assessment
ROC: receiver operating characteristic
AUC: Area under the receiver operating characteristic curve
VR: virtual reality
VSP: Virtual Supermarket Program
