# Supplementary figures and images for: A Virtual Supermarket Program for the Screening of Mild Cognitive Impairment in Older Adults: Diagnostic Accuracy Study
Source: JMIR Serious Games. 2021 Dec 3;9(4):e30919. doi: 10.2196/30919 (PMC8686451; doi:10.2196/30919)

**Multimedia Appendix 1：**

**Fig. a.** Navigation interface.

**
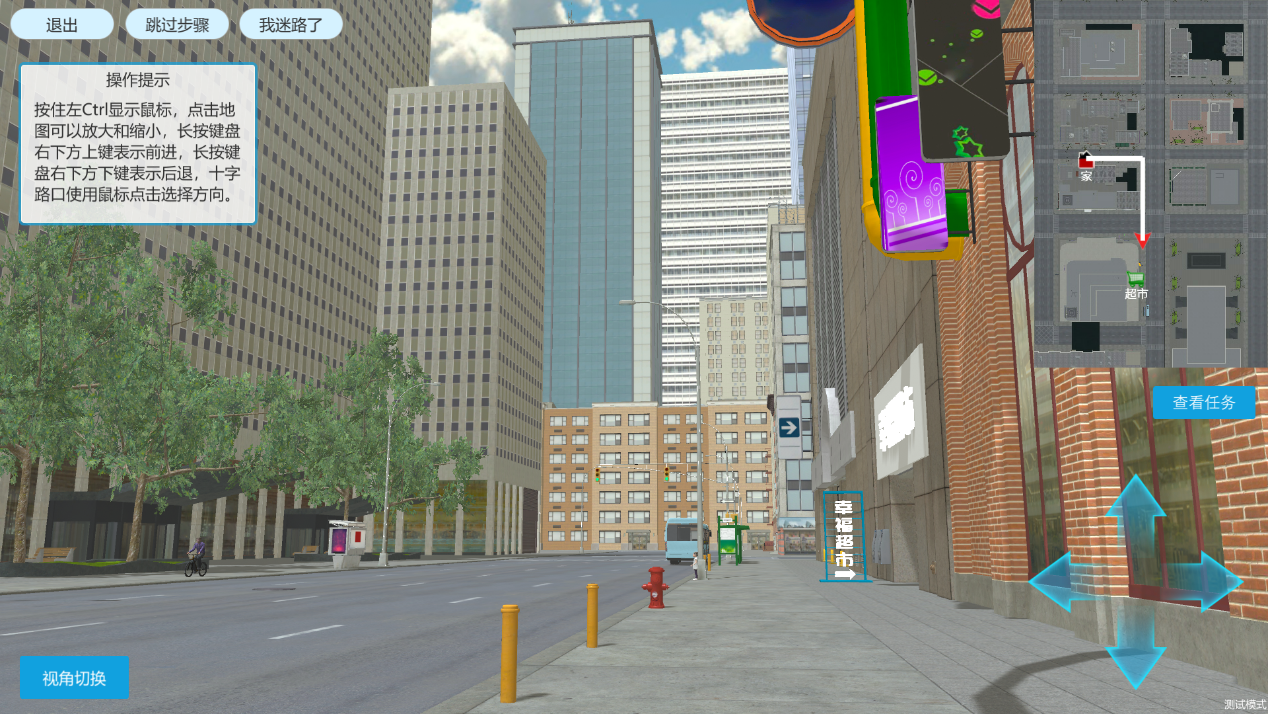
**

**Fig. b.** Shopping scene.

**
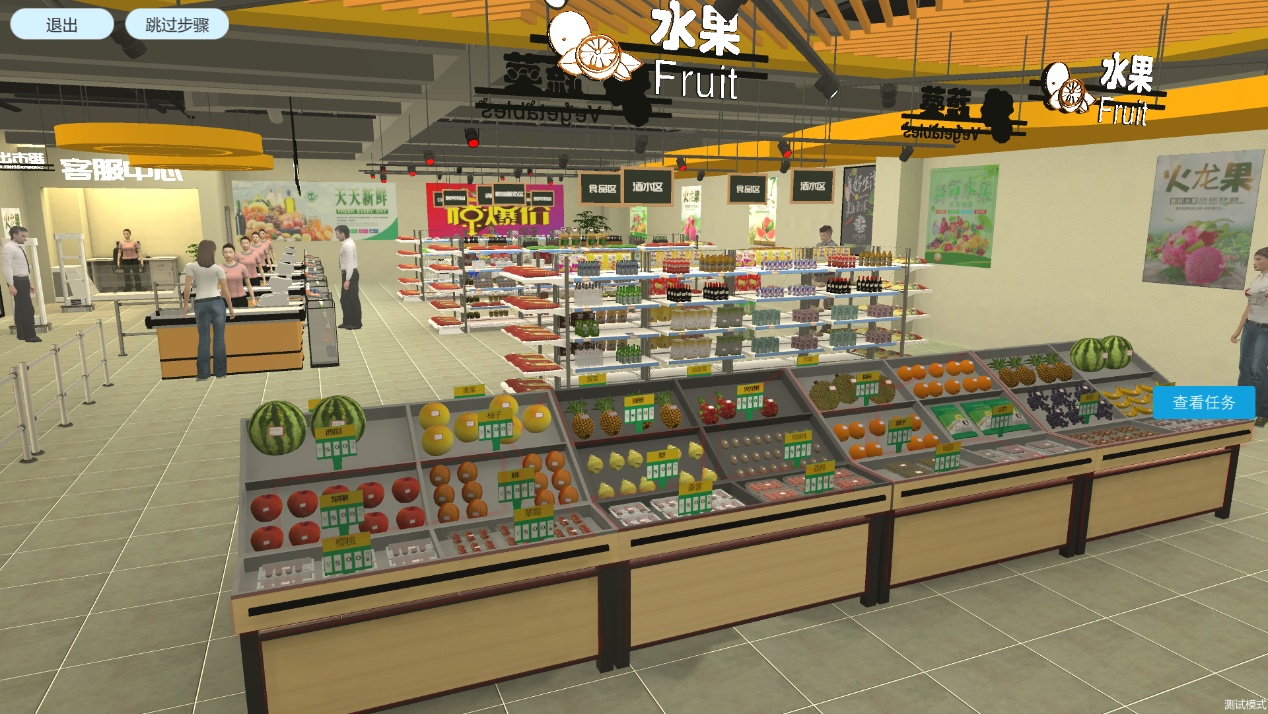
**

**
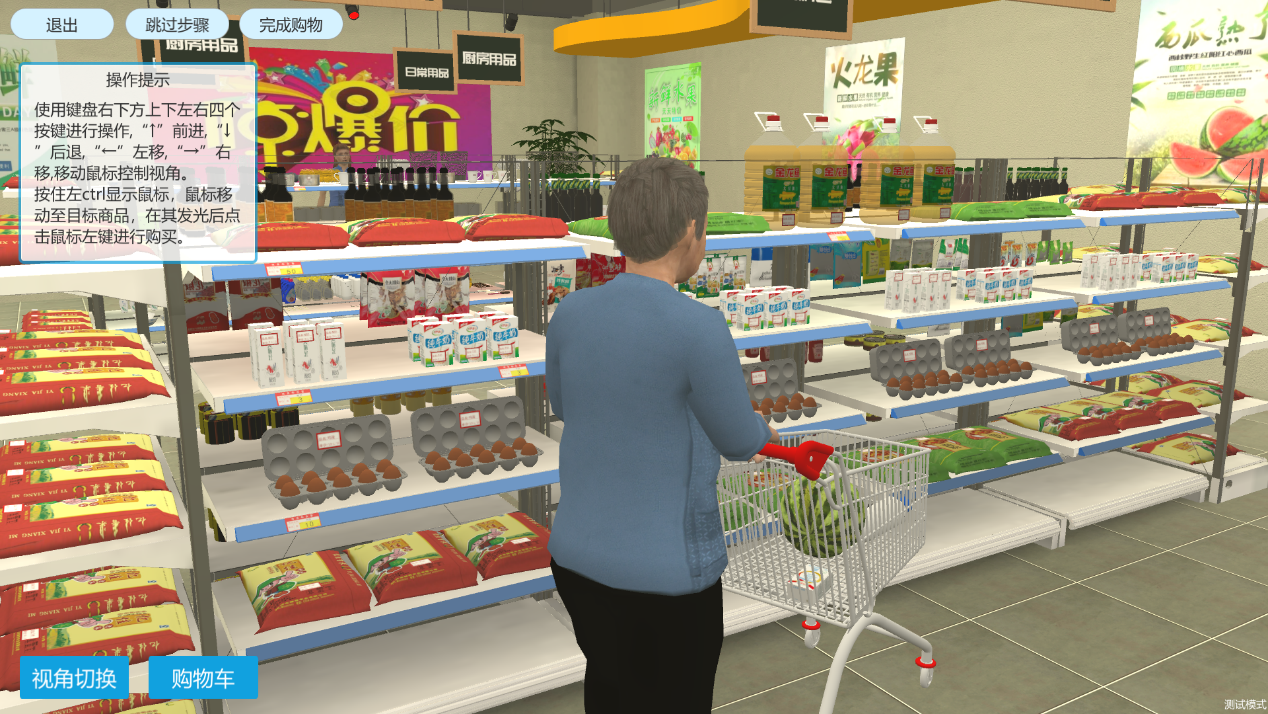
**

**Fig. c.** Rotate 360° to view items

**
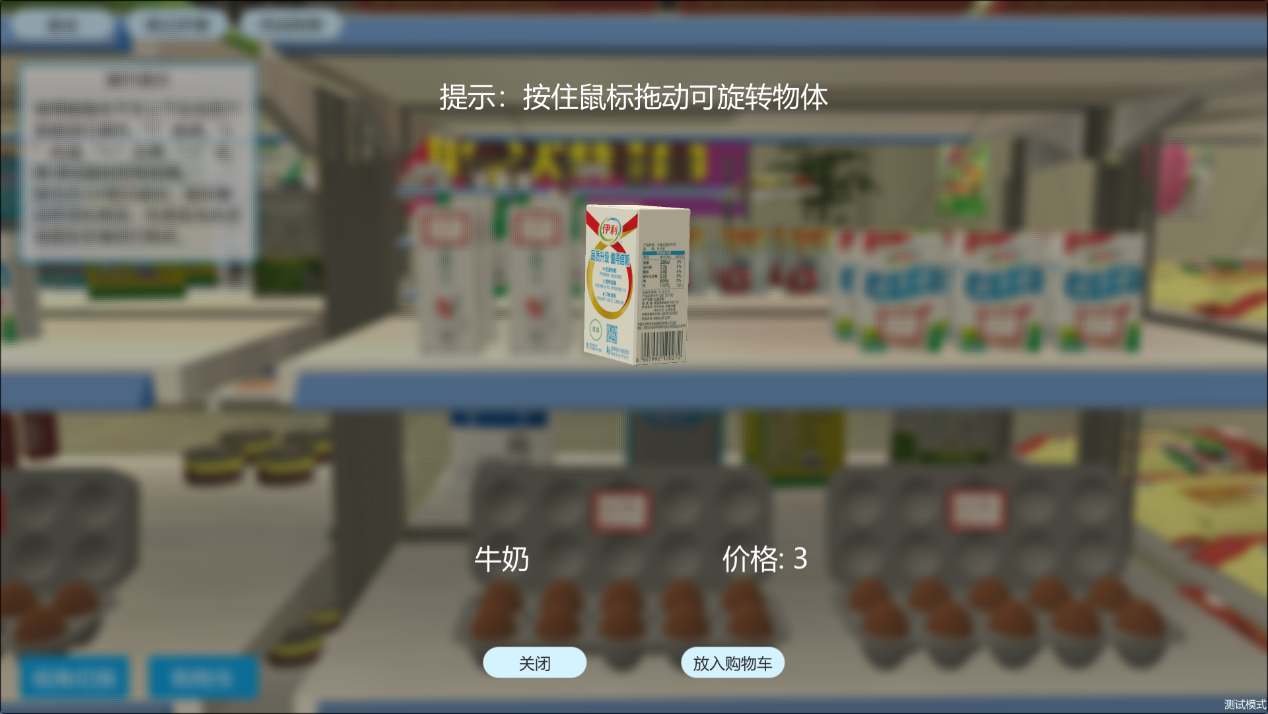
**

**
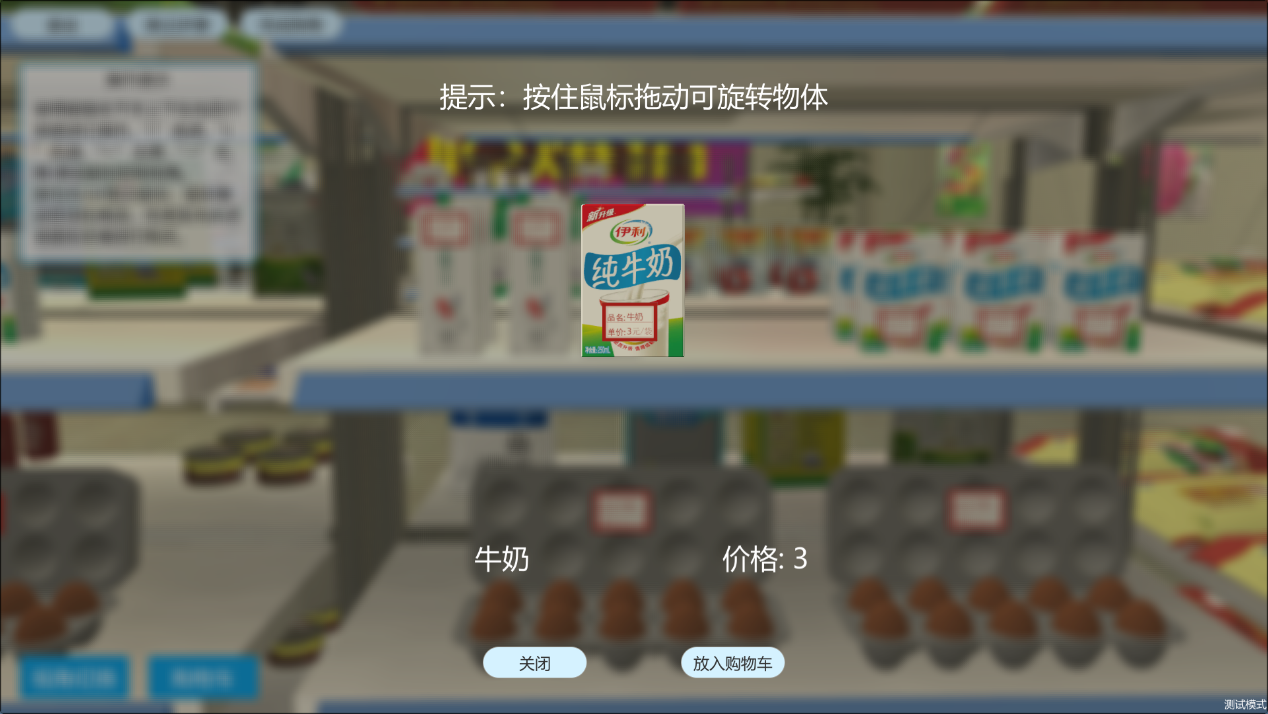
**

Supplement: Multimedia Appendix 1 [file games_v9i4e30919_app1.doc]
