# Supplementary material for: A Virtual Supermarket Program for the Screening of Mild Cognitive Impairment in Older Adults: Diagnostic Accuracy Study
Source: JMIR Serious Games. 2021 Dec 3;9(4):e30919. doi: 10.2196/30919 (PMC8686451; doi:10.2196/30919)
Supplement: Multimedia Appendix 2 [file games_v9i4e30919_app2.doc]

Multimedia Appendix 2: VSP score of each task and total score.

| Tasks | Score |
| --- | --- |
| Task 1 | 10 |
| Task 2 | 5 |
| Task 3 | 10 |
| Task 4 | 6 |
| Task 5 | 6 |
| Task 6 | 6 |
| Task 7 | 6 |
| Task 8 | 6 |
| Task 9 | 5 |
| Total | 60 |
